# Echinacoside Isolated from *Cistanche tubulosa* Putatively Stimulates Growth Hormone Secretion via Activation of the Ghrelin Receptor

**DOI:** 10.3390/molecules24040720

**Published:** 2019-02-17

**Authors:** Chieh-Ju Wu, Mei-Yin Chien, Nan-Hei Lin, Yi-Chiao Lin, Wen-Ying Chen, Chao-Hsiang Chen, Jason T. C. Tzen

**Affiliations:** 1 Graduate Institute of Biotechnology, National Chung-Hsing University, Taichung 402, Taiwan; baby159357520@gmail.com (C.-J.W.); CMNHEI@mohw.gov.tw (N.-H.L.); s9755702@gmail.com (Y.-C.L.); 2 Ko Da Pharmaceutical Co. Ltd., Taoyuan 324, Taiwan; rd1@koda.com.tw; 3 Department of Veterinary Medicine, National Chung-Hsing University, Taichung 402, Taiwan; wychen@dragon.nchu.edu.tw; 4 Graduate Institute of Pharmacognosy, Taipei Medical University, Taipei 110, Taiwan

**Keywords:** cistanche tubulosa, echinacoside, ghrelin, growth hormone secretion, phenylethanoid glycosides

## Abstract

*Cistanche* species, the ginseng of the desert, has been recorded to possess many biological activities in traditional Chinese pharmacopoeia and has been used as an anti-aging medicine. Three phenylethanoid glycosides—echinacoside, tubuloside A, and acteoside—were detected in the water extract of *Cistanche tubulosa* (Schenk) R. Wight and the major constituent, echinacoside, was further purified. Echinacoside of a concentration higher than 10^−6^ M displayed significant activity to stimulate growth hormone secretion of rat pituitary cells. Similar to growth hormone-releasing hormone-6, a synthetic analog of ghrelin, the stimulation of growth hormone secretion by echinacoside was inhibited by [D-Arg^1^, D-Phe^5^, D-Trp^7,9^, Leu^11^]-substance P, an inverse agonist of the ghrelin receptor. Molecular modeling showed that all the three phenylethanoid glycosides adequately interacted with the binding pocket of the ghrelin receptor, and echinacoside displayed a slightly better interaction with the receptor than tubuloside A and acteoside. The results suggest that phenylethanoid glycosides, particularly echinacoside, are active constituents putatively responsible for the anti-aging effects of *C. tubulosa* and may be considered to develop as non-peptidyl analogues of ghrelin.

## 1. Introduction

*Cistanche* species (Orobanchaceae), the so-called ginseng of the desert, is a holoparasitic plant found in the desert region of northwestern China. This herbal medicine has been recorded to possess several biological activities such as constipation relief, longevity, and aphrodisiac properties in traditional Chinese pharmacopoeia [1]. Different species of *Cistanche* are commonly named according to their hosts and growing environments, such as *Cistanche deserticola* Y.C. Ma and *Cistanche tubulosa* (Schenk) R. Wight. *C. deserticola*, hosted by *Haloxylon ammodendron* mainly found in Inner Mongolia, was originally regarded as the standard *Cistanche* source in Chinese Pharmacopoeia, but has been listed as an endangered plant species in China since 1984. *C. tubulosa*, hosted by *Tamarix ramosissima* grown in Xinjiang Hetian, tends to possess a fast growth rate, and thus agricultural cultivation of this species leads to a much better yield than the traditional *C. deserticola*. Nowadays, both of the above two species are collected in the Chinese Pharmacopoeia [2].

*C. deserticola* and *C. tubulosa* are found to possess similar chemical constituents and pharmacological activities, thus both of them are regarded to be potential herbal medicine for aging-related diseases [3,4,5]. Phenylethanol glycosides are common active ingredients found in these two species, and echinacoside is identified as the major phenylethanol glycoside in *C. tubulosa* [6]. In the past decade, echinacoside and the extract of *C. tubulosa* rich in echinacoside were demonstrated to possess many pharmacological activities, such as anti-aging, neuroprotection, enhancement of learning and memory, improvement of cardiac function, reduction of hyperlipidemia and hyperglycemia, and prevention of obesity-induced diabetes and metabolic syndrome [7,8,9,10,11,12].

Known as the “hunger hormone”, ghrelin is a peptide hormone composed of 28 amino acid residues in which the third serine residue is acylated with an n-octanoyl group essential for its biological functions [13]. Ghrelin has been demonstrated to be an endogenous ligand for a G-protein-coupled receptor named growth hormone secretagogue-1a receptor (GHS-R1a), which is mainly expressed in the hypothalamus and the pituitary gland [14]. Activation of GHS-R1a by ghrelin results in the stimulation of growth hormone secretion and the regulation of several physiological reactions [15]. Clinical studies show that exogenous supplementation with growth hormones could alleviate the symptoms of aging-related diseases in the elderly, such as increasing muscle strength and bone density and improving sleeping quality and cognitive ability [16,17]. However, concerns of serious side effects (e.g., prostate cancer, joint pain, insulin resistance, fluid retention, carpal tunnel syndrome, and gynecomastia) have been brought up for the treatment with exogenous growth hormones [18,19]. Therefore, the stimulation of growth hormone secretion by ghrelin via GHS-R1a activation has been considered as an alternative therapeutic approach for the aging-related diseases of the elderly, and its therapeutic effect is assumed to be superior to exogenous supplementation of growth hormone.

Recently, teaghrelins and ginkgoghrelins, unique acylated flavonoid glycosides found in Chin-shin oolong tea and *Ginkgo biloba* L. (Ginkgoaceae) leaves, were demonstrated to induce growth hormone secretion of rat primary anterior pituitary cells via binding to the ghrelin receptor [20,21,22]. Considering the therapeutic effects of *Cistanche* spp. on aging-related diseases were similar to the anti-aging effects of Chin-shin oolong tea and *Ginkgo biloba* leaves, we wondered whether the pharmacological activities of phenylethanol glycosides could be executed via the same pathway as those of teaghrelins and ginkgoghrelins by binding to the ghrelin receptor. Firstly, three phenylethanoid glycosides—echinacoside, tubuloside A, and acteoside—were identified in the water extract of *C. tubulosa*. The major constituent, echinacoside, was further purified and examined for its capability for stimulating growth hormone release of rat pituitary cells via activation of the ghrelin receptor. Molecular modeling of echinacoside, tubuloside A, and acteoside docking to the ghrelin receptor was performed and compared for the possible interactions within the binding pocket.

## 2. Results

### 2.1. Separation and Identification of Three Major Phenylethanol Glycosides in the Water Extract of Two Cistanche Species

**Figure 1:**
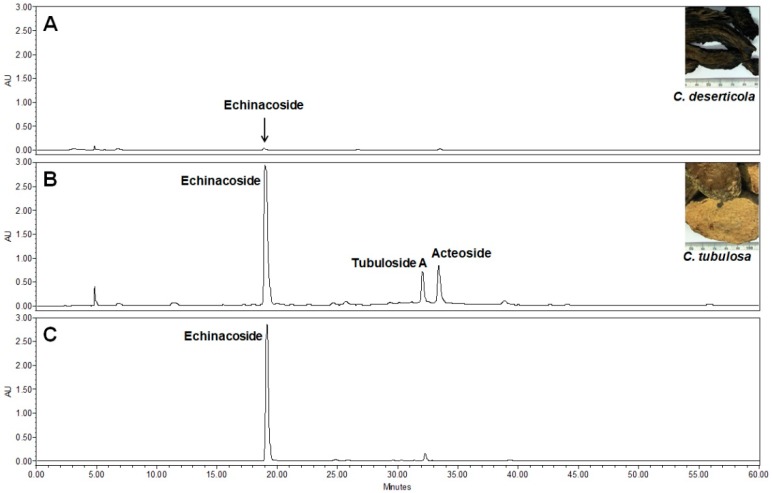
HPLC profiles of the water extract from *Cistanche deserticola* (**A**), the water extract from *C Cistanche tubulosa* (**B**), and isolated echinacoside (**C**) at 330 nm. The chemical constituents in the water extracts were separated by HPLC (0–60 min). The peaks of echinacoside, tubuloside A, and acteoside were labeled.

**Figure 2:**
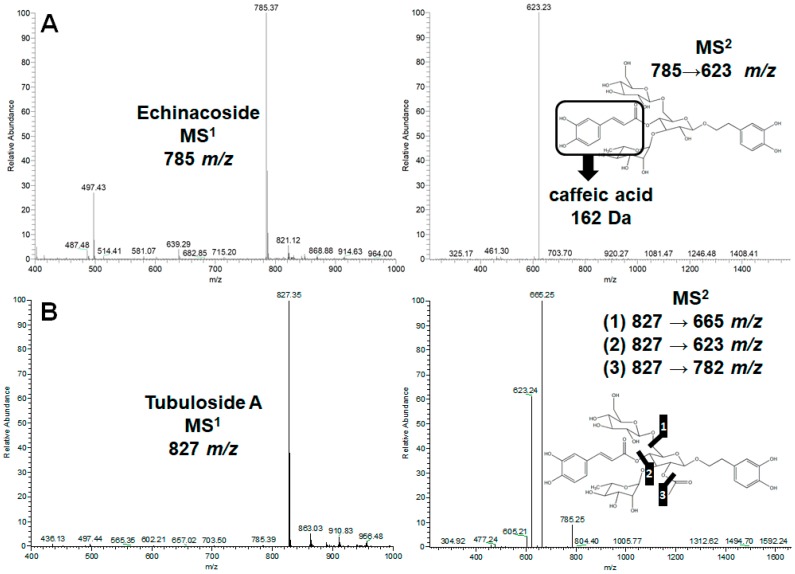

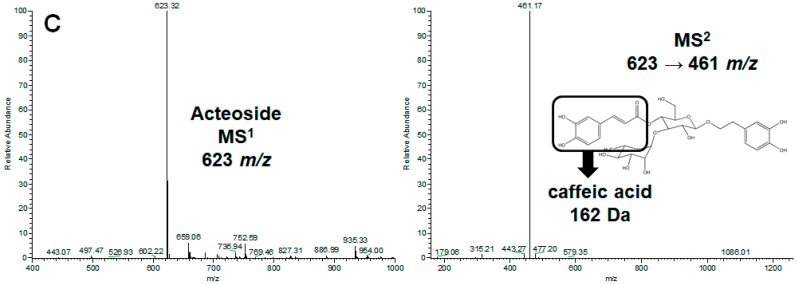
Mass spectrometric analyses of echinacoside (**A**), tubuloside A (**B**), and acteoside (**C**) in MS^1^ (left panels) and MS^2^ (right panels) fragmentation.

In the HPLC analysis of two *Cistanche* species, *C. tubulosa* was found to possess much higher contents of phenylethanol glycosides than *C. deserticola* (Figure 1A,B). Three major phenylethanol glycosides were observed in *C. tubulosa* and identified as echinacoside, tubuloside A, and acteoside by mass spectrometric analysis (Figure 2) in comparison with the HPLC profiles and MS data of known compounds published previously [23,24]. According to the molecular fragmentation 785 *m*/*z* in MS^1^ and 623 *m*/*z* in MS^2^, the major peak in the water extract of *C. tubulosa* was identified as echinacoside (Figure 2A). Similarly, tubuloside A was assigned by its molecular fragmentation 827 *m*/*z* in MS^1^ and 665, 623, and 782 *m*/*z* in MS^2^ (Figure 2B); acteoside was confirmed by the molecular fragmentation 623 *m*/*z* in MS^1^ and 461 *m*/*z* in MS^2^ (Figure 2C). Echinacoside was further purified to near homogeneity (Figure 1C) and used in the following biological assays.

### 2.2. Effect of Echinacoside on the Induction of Growth Hormone Secretion in Rat Pituitary Cells

**Figure 3:**
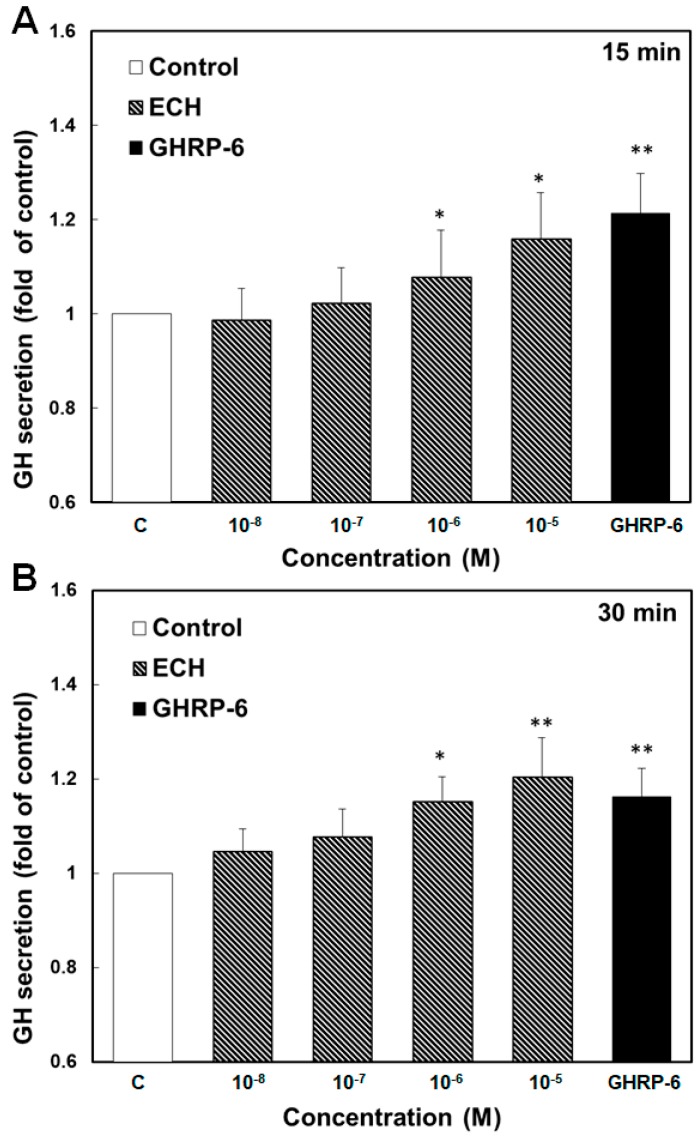
Effect of echinacoside administration on growth hormone secretion of pituitary cells. Growth hormone (GH) secretion from rat primary pituitary cells was measured after incubation with medium (control), GHRP-6 (GHSR agonist, 10^−7^ M), and various concentrations (from 10^−8^ to 10^−5^ M) of echinacoside (ECH) for 15 (**A**) and 30 min (**B**), respectively. Data were presented as means ± SD with *n* = 6. Significance levels seen by T Test were * *p* < 0.05 and ** *p* < 0.005 versus the control.

**Figure 4:**
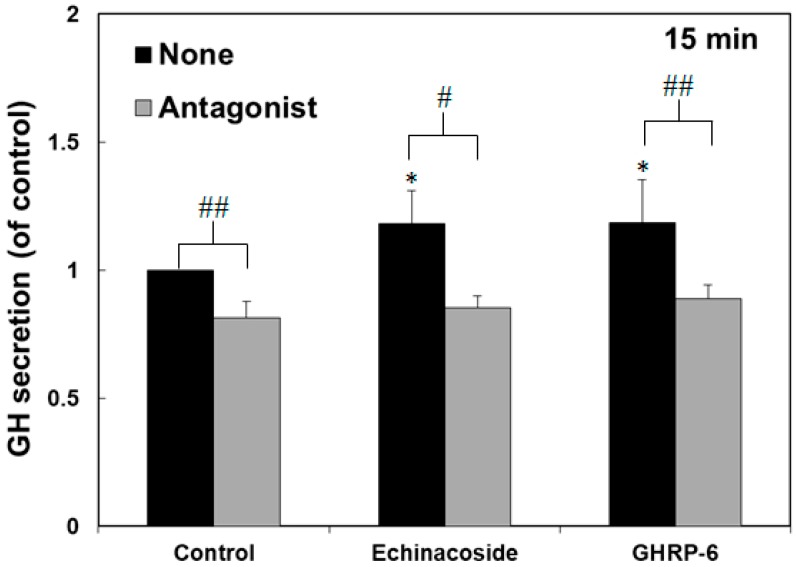
Inhibition of [D-Arg^1^, D-Phe^5^, D-Trp^7,9^, Leu^11^]-substance P (GHSR antagonist) on stimulatory effects of GH secretion by GHRP-6 and echinacoside treatment. Rat primary anterior pituitary cells were incubated with GHRP-6 (10^−7^ M) and echinacoside (10^−5^ M) in the presence and absence of [D-Arg^1^, D-Phe^5^, D-Trp^7,9^, Leu^11^]-substance P (0.5 μM) for 15 min. Data were presented as means ± SD with *n* = 6. Significance levels seen by T Test were * *p* < 0.05 versus the control, ^#^ *p* < 0.05 and ^##^ *p* < 0.005 significant differences between columns.

To evaluate if echinacoside could be an agonist of growth hormone secretagogue receptor (GHSR), it was subjected to examination of the induction of growth hormone secretion from primary rat anterior pituitary cells. Similar to GHRP-6, a synthetic GHSR agonist, echinacoside was able to stimulate the secretion of growth hormones from rat pituitary cells (Figure 3). Significant increases in growth hormone secretion were detected when rat pituitary cells were treated with echinacoside of a concentration higher than 10^−6^ M for 15 and 30 min. The treatment of echinacoside (from 10^−8^ to 10^−5^ M) for 15 and 30 min was found to stimulate growth hormone secretion of the rat pituitary cells in a dose dependent manner. Moreover, a GHSR inverse agonist, [D-Arg^1^, D-Phe^5^, D-Trp^7,9^, Leu^11^]-substance P, obviously inhibited the stimulatory effects of GHRP-6 and echinacoside on the induction of growth hormone secretion from rat pituitary cells (Figure 4). The results suggest that echinacoside is a potential agonist of GHSR.

### 2.3. Molecular Docking of Echinacoside, Tubuloside A, and Acteoside to the Ghrelin Receptor 

**Figure 5:**
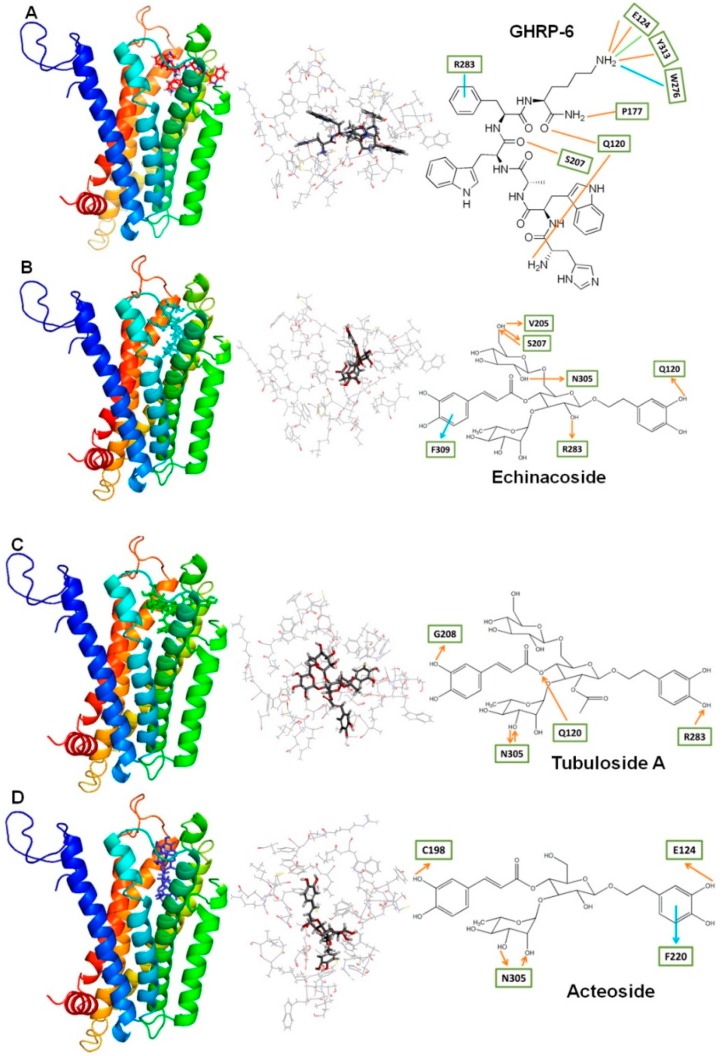
Modeling of four ligands, GHRP-6 (**A**), echinacoside (**B**), tubuloside A (**C**), and acteoside (**D**) docking into GHSR, and the detailed intermolecular interaction in these complex structures. Left panel: ligands (ball-stick structure) in the ghrelin binding site of GHSR (ribbon structure). Middle panel: the amino acids (stick structure) close to ligands (ball-stick structure) in these complex structures. Right panel: detailed intermolecular interaction between ligands and GHSR. The amino acids of GHSR involved in formation of interaction with ligands are shown in squares. Distances of H-bonding (red lines), charge-charge (green line), and π-π interaction (blue lines) are indicated.

In comparison with GHRP-6, the three phenylethanol glycosides in *C. tubulosa*—echinacoside, tubuloside A, and acteoside—were subjected to molecular modeling and docking to the binding pocket of the ghrelin receptor, GHSR. The results showed that all three phenylethanol glycosides could bind to the ghrelin binding pocket of GHSR, though their interactions within the binding pocket were relatively weak in comparison with GHRP-6 (Figure 5A). Two types of intermolecular interaction, H-bonding and π-π stacking, were formed between echinacoside and GHSR (Figure 5B). Six H-bonds were formed between echinacoside and GHSR; two between the C6 hydoxyl group of sugar moiety and S207 of GHSR, one between the C6 hydoxyl group of sugar moiety and V205 of GHSR, one between the C2 hydroxyl group of sugar moiety and N305 of GHSR, one between the C2 hydroxyl group of glycoside moiety and R283 of GHSR, and one between the C3 hydroxyl group of dihydroxyphenylethanol moiety and Q120 of GHSR. One π-π stacking interaction was formed between the caffeic acid of echinacoside and F309 of GHSR. Only one type of intermolecular interaction, H-bonding, was formed between tubuloside A and GHSR (Figure 5C). Five H-bonds were formed between tubuloside A and GHSR; two between the C3 hydoxyl group of rhamnose moiety and N305 of GHSR, one between the hydoxyl group of caffeic acid moiety and G208 of GHSR, one between the hydroxyl group of dihydroxyphenylethanol moiety and R283 of GHSR, and one between the C4 of glycoside moiety and Q120 of GHSR. Two types of intermolecular interactions, H-bonding and π-π stacking, were formed between acteoside and GHSR (Figure 5D). Four H-bonds were formed between acteoside and GHSR; two between the C2 and C3 hydoxyl groups of rhamnose moiety and N305 of GHSR, one between the hydoxyl group of caffeic acid moiety and C198 of GHSR, and one between the hydroxyl group of dihydroxyphenylethanol moiety and E124 of GHSR. One π-π stacking interaction was formed between the dihydroxyphenylethanol moiety of acteoside and F220 of GHSR. Accordingly, binding affinities (chemical energy) calculated by GEMDOCK showed that echinacoside, tubuloside A, and acteoside possessed comparable strength of ligand-receptor interaction, while echinacoside displayed a slightly better interaction with the receptor than tubuloside A and acteoside when docking to the binding pocket of the ghrelin receptor constructed with the pocket space occupied by GHRP-6 (Table 1).

## 3. Discussion

In literature, ghrelin (known as an endogenous hormone) and echinacoside found as a major phenylethanoid glycoside in *Cistanche* species were documented to possess several equivalent physiological activities, including anti-aging effects presumably through hypothalamus-pituitary-gonadal axis. Indeed, both ghrelin and echinacoside were proposed to be therapeutic drugs in Alzheimer’s disease and Parkinson’s disease [3,4,8,25,26,27]. In this study, we showed that echinacoside isolated from *C. tubulosa* might stimulate growth hormone secretion from rat primary anterior pituitary cells putatively via GHSR activation in a manner similar to ghrelin. The stimulation of growth hormone secretion triggered by echinacoside seems to support the well-recognized anti-aging effects of *Cistanche* species, and phenylethanoid glycosides, particularly echinacoside, are active constituents putatively responsible for the therapeutic effects.

Estimated from the relative capability of stimulating growth hormone secretion from rat pituitary cells, echinacoside seemed to be approximately 100 times weaker than GHRP-6, as comparable stimulation activities were observed between echinacoside of 10^−5^ M and GHRP-6 of 10^−7^ M for treatments of 15 and 30 min (Figure 3). The relative activities of echinacoside and GHRP-6 in inducing growth hormone secretion were in good agreement with the molecular modeling, as GHRP-6 strongly interacted with the residues around the binding pocket of GHSR via three types of interaction—H-bonding, charge-charge, and π-π interaction—while echinacoside interacted with GHSR via two types of interaction—H-bonding and π-π stacking (Figure 5). Notably, it has been highlighted that the charge-charge interaction between the positively charged ammonium group of GHRP-6 and the negatively charged E124 of GHSR plays a critical role in the binding of GHRP-6 to the ghrelin receptor [28]. Possibly, the relatively weak stimulatory effect of echinacoside on the induction of growth hormone secretion from rat pituitary cells might be mainly attributed to the lack of charge-charge interaction in the binding pocket of GHSR.

According to the activity assay in this study (Figure 3), the minimal effective concentration of echinacoside (*Mr* 786) for the stimulation of growth hormone secretion from rat pituitary cells is around 10^−6^ M. For an adult of 65 kg, the blood volume is approximately 5 L (65 kg × 1/13 = 5 kg ≈ 5 L), and thus about 4 mg of echinacoside is required to reach a concentration of 10^−6^ M (5L × 10^−6^ M × 786 = 3.93 mg). Approximately 20 mg of echinacoside is extracted from 1 g of dried stem of *C. tubulosa* [24]. To obtain 4 mg of echinacoside, it requires 0.2 g of dried stem of *C. tubulosa*. Commonly, more than 1 g of dried stem of *C. tubulosa* is added in the preparation of medicinal products. Therefore, the effective dosage of echinacoside seems to be in good agreement with the empirical consumption of *C. tubulosa* as an anti-aging herbal medicine. Of course, the effective dosage of echinacoside should be higher than the above estimation, as the availability of echinacoside tends to be reduced after intestinal absorption and passing through the blood-brain barrier. Further experiments are required to exactly determine the effective dosage of echinacoside in animals.

In our previous studies, teaghrelin and ginkgoghrelin, unique acylated flavonoid glycosides isolated from Chin-shin oolong tea and *Ginkgo biloba* leaves, were also shown to induce growth hormone secretion of rat pituitary cells via binding to the ghrelin receptor [20,22]. Interestingly, the minimal effective concentration of either teaghrelin or ginkgoghrelin for the stimulation of growth hormone secretion from rat pituitary cells was also found to be around 10^−6^ M, and thus equivalent to that of echinacoside. However, the contents of teaghrelin and ginkgoghrelin in Chin-shin oolong tea and *Ginkgo biloba* leaves were relatively low; there was approximately 1 mg of teaghrelin in 1 g of Chin-shin oolong tea and 0.33 mg of ginkgoghrelin in 1 g of *Ginkgo biloba* leaves in contrast with the 20 mg of echinacoside in 1 g of *C. tubulosa.* Therefore, the effective consumption dosages of Chin-shin oolong tea and *Ginkgo biloba* leaves are around 5–10 g and 10–20 g, respectively. The drastic difference in effective consumption dosage between *C. tubulosa* (0.2 g) and Chin-shin oolong tea or *Ginkgo biloba* leaves seems to explain why *C. tubulosa* is used as an anti-aging herbal medicine while Chin-shin oolong tea and *Ginkgo biloba* leaves are consumed as herbal tea drinks. 

## 4. Materials and Methods

### 4.1. Chemicals and Plant Materials

*Cistanche deserticola* Y.C. Ma was obtained from a local market and authenticated by Dr. Nan-Hei Lin. *Cistanche tubulosa* (Schenk) R. Wight was purchased from Sinphar Tian-Li Pharmaceutical Co., Ltd., (Hangzhou, China). HPLC-grade acetonitrile, formic acid, and methanol were purchased from ECHO Chemical Co., Ltd, (Miaoli, Taiwan). Dulbecco’s modified Eagle’s medium (DMEM), dialyzed fetal bovine serum (DFBS), and Trypsin-EDTA were bought from Invitrogen (Carlsbad, CA, USA). DNase I was bought from Worthington Biochemical (Lakewood, NJ, USA). Growth hormone-releasing hormone-6 (GHRP-6) was obtained from Gen Way Biotech, Inc. (San Diego, CA, USA). Collagenase type I and [D-Arg^1^, D-Phe^5^, D-Trp^7,9^, Leu^11^]-substance P were bought from Sigma-Aldrich Co. (St. Louis, MO, USA). Rat growth hormone ELISA kit was purchased from Sunred Biological Technology Corporation (Shanghai, China).

### 4.2. HPLC/UV and LC−MS^n^ Analyses of the Water Extraction of Cistanche spp.

Dried stem (25 g) of *Cistanche deserticola* Y.C. Ma or *Cistanche tubulosa* (Schenk) R was extracted three times with 500 mL distilled water for 60 min at 50 °C in a water bath. The solution was filtered through a 13 mm Syrign Filter with 0.45 μm PP membrane filter (Pall Corporation, Glen Cove, NY, USA), and subjected to the following analyses. The chemical constituents in the extracts were analyzed using a Syncronis C18 column (4.6 × 250 mm inner diameter, 5 μm, Thermo Scientific, Waltham, MA, USA) in the HPLC system coupled with a model 600E photodiode array detector (Waters Corporation, Milford, MA, USA). The mobile phase consisted of (A) water containing 0.1% formic acid and (B) acetonitrile. The eluting gradient was as follows: 0–60 min, linearly gradient from 14% B; 0–3 min, 14% to 17% B; 3–4 min, 17% B; 4–15 min, 17% to 20% B; 15–20 min, 20% B; 20–50 min, 20% to 14% B; 50–60 min, 14% B. The ultraviolet (UV) absorbance detection wavelength was set at 330 nm. A linear trap quadrupole (LTQ) tandem mass spectrometer (Thermo Electron, San Jose, CA, USA) equipped with an electrospray ionization (ESI) interface were connected to a Surveyor LC system (Thermo Electron) with a 5 μL sample loop. The eluting gradient was as follows: 0–90 min, linearly gradient form 14% B; 0–24 min, 14% to 17% B; 24–25 min, 17% B; 25–36 min, 17% to 20% B; 36–37 min, 20% B; 37–80 min, 20% to 14% B; 80–90 min, 14% B. The heated capillary temperature was set at 300 °C with the spray voltage of 4.5 kV. Negative ESI mode was firstly scanned ranging from *m*/*z* 400–1000. Data-dependent MS^n^ was obtained using the high purity helium (>99.99%) as the collision gas. 

### 4.3. Isolation of Echinacoside 

The water extract from dried stem (25 g) of *C. tubulosa* was concentrated under reduced pressure to give deep brown syrup. The crude extraction was suspended with distilled water and lyophilized by a freeze dryer. The powder of 100 mg was resolved in distilled water of 5 mL and subjected to purification using Sephadex LH-20 column (100 mL; GE Healthcare Bio-Sciences AB, Sweden) eluted with 10% aqueous methanol solution and monitored by HPLC. The fractions containing echinacoside were detected by reading the absorbance at 245 nm and harvested by using an autosampler. 

### 4.4. Animals

The experiments were approved by the Institutional Animal Care and Use Committee of the National Chung−Hsing University with the approval number of IACUC 106-079. Male Sprague−Dawley rats weighing 250−300 g were purchased from BioLASCO, Taiwan Co., Ltd. (Taipei, Taiwan). Two animals per cage were maintained in a controlled environment of 23 ± 2 °C, 60 ± 10% humidity, and 12 h light/dark cycle. The rats were fed with a standard chow diet (calories provided by 28.7% protein, 13.4% fat, and 57.9% carbohydrate, 5001 Rodent LabDiet, St. Louis, MO, USA) and distilled water ad libitum.

### 4.5. Primary Pituitary Cell Culture

Pituitary cells were isolated according to a modified enzymatic dispersion method developed by Yamazaki et al. [29]. Briefly, male Sprague Dawley rats were anaesthetized with Zoletil 50 (40 mg/kg, IP; Virbac Laboratories, Carros, France), and the anterior pituitary glands were removed and dispersed to culture pituitary cells in suspension, as described previously [30]. 

### 4.6. Growth Hormone Secretion Assay

The primary anterior pituitary cells of 4 × 10^4^ cell/well were cultured at 37 °C under 5% CO_2_ for 2 days prior to the growth hormone secretion assay according to the protocol described previously [30]. After the removal of culture medium, cells were starved in serum-free Dulbecco’s Modified Eagle’s medium (DMEM) for 90 min to stabilize basal hormone secretion. The starvation medium was replaced with fresh DMEM containing echinacoside (from 10^−8^ to 10^−5^ M) or GHRP-6 (agonist of a human ghrelin receptor, GHSR, 10^−7^ M) as a positive control, and cells were incubated for 15 and 30 min at 37 °C under 5% CO_2_. For detection of antagonist effect, the cells were incubated with a GHSR inverse agonist, [D-Arg^1^, D-Phe^5^, D-Trp^7,9^, Leu^11^]-substance P (0.5 μM), and then treated with DMEM containing echinacoside (10^−5^ M) or GHRP-6 (10^−7^ M) for 30 min. The medium was collected for determination of growth hormone secretion by a rat growth hormone ELISA kit (Shanghai Sunred Biological Technology Corporation).

### 4.7. Statistical Analysis

The data were presented as mean values ± SD. The differences were analyzed by T Test. Statistical calculations were performed by GraphPad Prism 6 (GraphPad Software Inc., La Jolla, CA, USA). A level of *p* < 0.05 was considered to be statistically significant.

### 4.8. Homology Modeling and Docking

Homology modeling and docking to a human ghrelin receptor, growth hormone secretagogue receptor (GHSR, accession number AAI13548), was established by following our previous construction [21,24]. Briefly, crystal structures of β1 and β2 adrenergic receptors (PDB 2YCY and 3PDS) with bound ligands, cyanopindolol, and FAUC50 were used as templates to construct the GHSR structure [31,32]. The GHSR structure with the lowest PDF total energy was selected for further docking with GHRP-6, echinacoside, tubuloside A, and acteoside. All modeling processes were performed using the Discovery Studio 2.1 platform (http://accelrys.com/).

The 3D structure of GHRP-6 was downloaded from the Pub-Chem compound database on the NCBI website (http://www.ncbi.nlm.nih.gov/). The 3D structures of echinacoside, tubuloside A, and acteoside were built using the Chem3D program (http://www.cambridgesoft.com/). The ligand binding site of GHSR was defined as the spherical space with a 14 Å radius from the center of the binding pocket in the docking simulation. Docking of GHRP-6, echinacoside, tubuloside A, or acteoside to the binding site of GHSR was performed in silico by employing the LibDock module in the Discover Studio 2.1 package and further minimized by smart minimize algorithm with CHARMm force field in the Discover Studio 2.1 package [33]. To compare the relative binding affinities of echinacoside, tubuloside A, and acteoside in GHSR, the active center area constructed with GHRP-6 in GHSR was used for the docking, and the binding energy was calculated by GEMDOCK (The Institute of Bioinformatics, National Chiao Tung University, Taiwan).

## Figures and Tables

**Table 1:** Chemical energy calculated by GEMDOCK for the interaction between the binding pocket of the ghrelin receptor (GHSR) and three phenylethanoid glycosides (echinacoside, tubuloside A, and acteoside).

Compound	Energy (kJ mol^−1^)	Van der Waals’ Force (kJ mol^−1^)	H Bond (kJ mol^−1^)
Echinacoside	−132.35	−111.82	−20.53
Tubuloside A	−122.88	−103.93	−18.95
Acteoside	−120.35	−93.65	−26.7
